# Cooccurrence of Mycotoxins in Maize and Poultry Feeds from Brazil by Liquid Chromatography/Tandem Mass Spectrometry

**DOI:** 10.1155/2013/427369

**Published:** 2013-11-14

**Authors:** Maria de Lourdes Mendes de Souza, Michael Sulyok, Otniel Freitas-Silva, Sônia Soares Costa, Catherine Brabet, Miguel Machinski Junior, Beatriz Leiko Sekiyama, Eugenia Azevedo Vargas, Rudolf Krska, Rainer Schuhmacher

**Affiliations:** ^1^EMBRAPA Food Technology, Avenida das Américas 29501, 23020-470 Rio de Janeiro RJ, Brazil; ^2^Núcleo de Pesquisas de Produtos Naturais, Universidade Federal do Rio de Janeiro, 21941-902 Rio de Janeiro RJ, Brazil; ^3^Center for Analytical Chemistry, Department for Agrobiotechnology, IFA-Tulln, University of Natural Resources and Applied Life Sciences, Konrad-Lorenz-Street 20, 3430 Tulln, Austria; ^4^CIRAD, Département PERSYST, UMR QualiSud, TA B-95/16, 73 rue Jean-François Breton, 34398 Montpellier Cedex 5, France; ^5^Department of Clinical Analysis, Toxicology Laboratory, State University of Maringá, Colombo Avenue 5790, 87020-900 Maringá, Brazil; ^6^MAPA, Avenida Raja Gabaglia 245, Cidade Jardim, 30380-090 Belo Horizonte, MG, Brazil

## Abstract

The objective of this study was to quantitatively evaluate mycotoxins in samples of maize and poultry feed produced in Brazil. A multimycotoxin method based on HPLC-MS/MS was applied to investigate the occurrence of toxical fungal metabolites in 119 samples collected from poultry feed factory integrated poultry farms: maize grain (74), poultry feed (36), and feed factory residue (9). Twenty of 101 fungal metabolites investigated were detected and quantified in the samples: aflatoxins B_1_, B_2_, G_1_, and G_2_, fumonisins B_1_, B_2_, and B_3_, hydrolyzed fumonisin B_1_, zearalenone, agroclavine, chanoclavine, deoxynivalenol, and nivalenol, and enniatin A, A_1_, B, B_1_, beauvericin, kojic acid, and moniliformin. Most samples were contaminated with more than one mycotoxin. All samples were contaminated with fumonisins, with medians values of 1,840 **μ**g/kg, 239 **μ**g/kg, and 23,676 **μ**g/kg for maize, feed, and factory residue samples, respectively. Surprisingly, beauvericin was detected in more than 90% of samples. The median contaminations of aflatoxin and trichothecenes were low, near LOD values. The factory residue presented highest contamination levels for all mycotoxins. This is the first study dealing with agroclavine, chanoclavine, enniatin A, A_1_, B, B_1_, beauvericin, and kojic acid contamination of maize and poultry feeds from Brazil.

## 1. Introduction

Brazil is the third major maize producer country of the world after United States and China. In particular, in 2012, it produced 71.5 million tons [1] which represents about 8.31% of the total world production [2]. Maize is produced all over the country; nevertheless, more than half of the national production is concentrated in three states. Considering the Brazilian production in 2012, Paraná is the major producer state with 23.4%, followed by Mato Grosso (21.9%) and Goias (11.5%). Brazilian maize production is destined mainly for animal feeding (82%) especially for poultry and pigs production [3].

The infection of cereal crops by phytopathogenic *Fusarium* fungi in the field as well as by fungi of the genera *Aspergillus* and *Penicillium* during processing and storage leads to the contamination of the food chain by toxic secondary fungal metabolites, the mycotoxins [4]. The most common mycotoxins in cereals are the *Fusarium* mycotoxins deoxynivalenol (DON), zearalenone (ZEA), and the fumonisins (FUM) and *Penicillium* and *Aspergillus* mycotoxins ochratoxin A (OTA) and aflatoxins (AFs) [5]. Toxicity, metabolism and impact of these mycotoxins on human and animal health are already well-known and were subject for many reviews [6–9].

Research efforts to establish the magnitude of the mycotoxin occurrence in Latin America were initiated in the late 1960s after the outbreak of Turkey X disease. The bulk of mycotoxin research in Latin America has been conducted on maize and specifically on aflatoxins, although other toxins such as zearalenone, T-2 toxin, DON, penicillic acid, kojic acid, and ochratoxin have been detected in that cereal [10]. Recently, the Brazilian Regulation has changed including other mycotoxins beyond aflatoxins as well as decreasing the maximum tolerable levels for many commodities, especially for children's feed [11].

According to Salay and Zerlotti Mercadante [12], the incidence of aflatoxins, ochratoxin A, and zearalenone in maize cultivated in São Paulo State was much lower than the one from the northern and southern states. However, the incidence of fumonisins in maize seems to be widespread all over Brazil. Although the number of samples analyzed was small, the contamination of moniliformin, cyclopiazonic acid, sterigmatocystin, deoxynivalenol, and toxin T-2 seemed to not be relevant in Brazilian maize. The maize companies and feed industries consider expensive costs for the control of mycotoxins; therefore, few of them monitor other toxins than aflatoxins.

In a review dealing with mycotoxin research in Brazil between 1991 and 2000, Rodriguez-Amaya and Sabino [13] observed that thirty percent of the published articles surveyed mycotoxins in foods and feeds. AF occurrence in maize was low and occasional. As in other parts of the world, including other countries in Latin American, high contamination of maize and maize-based products with fumonisins (FBs) is widespread. Contamination with other mycotoxins, such as zearalenone (ZON), ochratoxin A (OTA), and trichothecenes, is low.

The results from another study indicate a low occurrence of trichothecenes mycotoxins in maize-based products commercialized in the city of São Paulo in spite of high levels of T-2 and HT-2 toxins found in one sample and show no immediate cause of concern. Nonetheless, more extensive surveys conducted for several years are advisable in order to furnish a more complete picture of the incidence of these toxins as well as other eventual (emergent) toxins in Brazilian products [14].

Maize is the major crop frequently exposed to the risk of contamination by all these mycotoxins. In particular, for maize, the European Commission has established maximum permitted levels for aflatoxins (AFB_1_, 2 *μ*g/kg; total AFs, 4 *μ*g/kg), OTA (5 *μ*g/kg), ZON (100 *μ*g/kg), and DON (1250 *μ*g/kg); FBs (2000 *μ*g/kg, FB_1_+ FB_2_) and limits for T-2 and HT-2 toxins are currently under discussion [15].

While most screening methods for mycotoxins addressed by legislation are based on immunoassays, unambiguous analytes confirmation can be easily achieved with mass spectrometric methods, such as gas chromatography/mass spectrometry (GC/MS) or liquid chromatography/mass spectrometry (LC/MS). During the last few years, this technical and instrumental progress had also an increasing impact on the expanding field of mycotoxin analysis [16]. The development of multimycotoxin methods [17–19] enables analyzing a larger fraction of the 300–400 fungal metabolites which are currently recognized as mycotoxins.

The present work aimed to investigate mycotoxin contamination in a poultry maize-based feed chain in Brazil by using a HPLC-MS/MS multimycotoxin method.

## 2. Materials and Methods

### 2.1. Chemicals and Reagents

Methanol and acetonitrile (both LC gradient grade) were purchased from J.T. Baker (Deventer, The Netherlands) and ammonium acetate (MS grade) and glacial acetic acid (p.a.) from Sigma-Aldrich (Vienna, Austria). Water was purified successively by reverse osmosis and a Milli-Q plus system from Millipore (Molsheim, France). Details concerning standards of the investigated mycotoxins (which include trichothecenes, zearalenone derivatives, fumonisins, ergot alkaloids, aflatoxins, ochratoxins, and some other metabolites produced by *Aspergillus* and *Penicillium* species) are described by Sulyok et al. [19].

### 2.2. Collection of Samples

A total of 119 samples of maize grains, subproducts, and poultry feeds were collected from a poultry feed factory and integrated poultry farms in Paraná State, in Brazil, from 2005 to 2006. The samples obtained were as follows: (i) 74 samples of maize grains were randomly withdrawn from trucks (from each truck one sample of 10 kg) in the poultry feed factory reception and factory processing steps (3 kg); (ii) 36 samples of poultry feeds (3 kg) in the integrated poultry farms; and (iii) 9 samples of maize factory residues (10 kg each) collected in the discarding of first cleaning (after sieving).

All samples were ground in a TREU mill (7.5 CV, 1720 rpm) with a 20 mesh sieved at Embrapa Food Technology, homogenized during 15 min (Chopin MR10L), packed under vacuum, and frozen stored until analyzed.

### 2.3. Sample Preparation and LC-MS/MS Determination

To 5 g of milled sample, 20 mL of extraction solvent (acetonitrile/water/acetic acid 79 : 20 : 1, v/v/v) was added. Extraction, dilution, and analysis were performed as described by Sulyok et al. [19]. Detection and quantification were performed with a QTrap 4000 LC-MS/MS System (Applied Biosystems, Foster City, CA) equipped with a Turbo Ion Spray electrospray.

Ionization (ESI) source and an 1100 Series HPLC System were brought from Agilent, Waldbronn, Germany. Chromatographic separation was performed at 25°C on a Gemini C18 column, 150 × 4.6-mmi.d., 5-*μ*m particle size, equipped with a C18 4 × 3-mm-i.d. security guard cartridge (all from Phenomenex, Torrance, CA, US). Both eluents contained 5 mM ammonium acetate and were composed of methanol/water/acetic acid 10 : 89 : 1 (v/v/v; eluent A) or 97 : 2 : 1 (eluent B), respectively. After an initial time of 2 min at 100% A, the proportion of B was increased linearly to 100% within 12 min, followed by a hold-time of 3 min at 100% B and 4-min column reequilibration at 100% A. The flow rate of 1 mL/min ESI-MS/MS was performed in the multiple reaction monitoring (MRM) modes both in positive and negative polarities in two separate chromatographic runs per sample by scanning two fragmentation reactions per analyte.

### 2.4. Recovery of Mycotoxins and Limits of Detection from Spiked Samples

The recovery was determined in duplicate by spiking in three different maize and feed sample. It spiked 0.5 g of sample in an open vial with appropriate amounts of a multianalyte working solution. The samples were subsequently stored for one day at room temperature to allow solvent evaporation. After this period, 2 mL of extraction solvent (acetonitrile/water/acetic acid 79 : 20 : 1, v/v/v) was added, and the same analytical procedure used as for the investigated samples was followed. Because all the investigated samples were naturally contaminated by fumonisins, the samples with the lowest levels were used for spiked experiments.

Limits of detection were calculated from the signal to noise ratios (LOD = 3 × S/N) of the respective multiple reaction monitoring (MRM) chromatograms deriving from the analysis of spiked samples.

## 3. Results and Discussion

The significance of mycotoxin contamination in food gained much attention over the past four decades. The cooccurrence of mycotoxins had been already described in maize and others foods [20–24]. It can affect both the level of mycotoxin production and the toxicity of the contaminated grains resulting in additive and synergistic effects. The surveillance of mycotoxins in maize is important for further toxicological studies especially for poultry industry that could indicate which toxins are relevant for further investigations.

Quantitative analysis of raw extracts by LC-MS/MS can be disturbed by signal suppression due to matrix effects. As these were investigated in maize only for a smaller set of 39 analytes [17], recovery tests were performed by spiking three individual samples of both matrices (maize and poultry feed). As we have previously observed that matrix effects may also vary between individual samples of a given matrix [19], three different samples per matrix were spiked. Table 1 lists the spiking levels, the limit of detection (LOD), and average recoveries of the investigated mycotoxins. In general, the values obtained for the apparent recoveries were in good agreement with the results obtained earlier [17, 18]. In that aspect, apparent recoveries significantly lower than 100% occurred for fumonisins (due to incomplete extraction), aflatoxins (due to matrix effects), ergot alkaloids (due to incomplete extraction and epimerization in case of ergopeptides—note the difference between -ines and -inines), and some other polar analytes. However, the apparent recoveries of some additional analytes (such as gliotoxin, chaetoglobosin A, and chaetomin) were unexpectedly low, which indicates that any findings concerning a specific matrix or analyte should not be overgeneralized. For example, the apparent recoveries in feed were slightly lower in comparison to those in maize for just a few analytes (e.g. fumonsins, aflatoxins B_2_, and G_2_), although the former matrix is considered to be far more complex. Nevertheless, it must be emphasized that matrix effects have to be carefully reevaluated for every analyte if the method is transferred to a new matrix. For most analytes, differences between the recoveries of individual samples of a given matrix were within the precision of the method.

**Figure 1:**
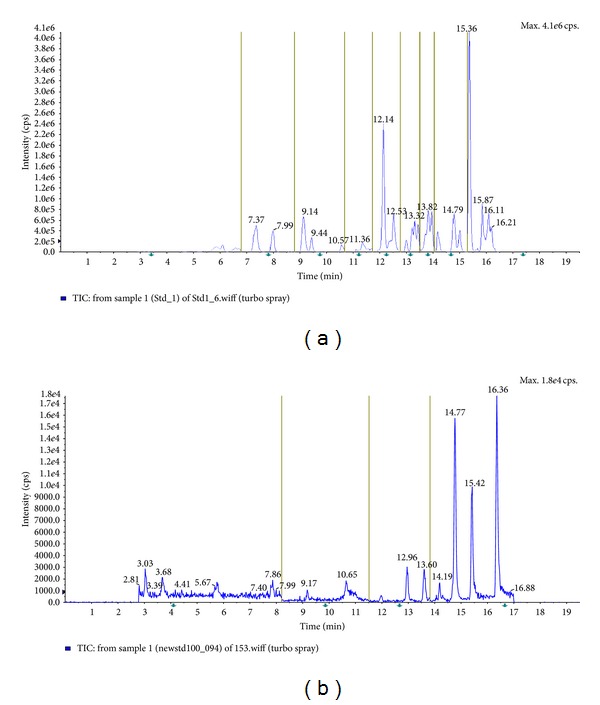
Total ions chromatogram (TIC) of 80 mycotoxins standards in positive mode (a) and of 21 mycotoxins standards in negative mode (b) obtained by HPLC-MS/MS (ESI).

Figure 1 shows total ions chromatogram (TIC) in positive and negative mode of 101 analcites analyzed by HPLC-MS/MS (ESI).

Table 2 gives the contamination range, median, and percentage of contaminated samples for each mycotoxin found in maize, poultry feed, and factory maize residue samples collected from the poultry feed factory reception and integrated poultry farms. All samples were contaminated with FB_1_, FB_2_, and FB_3_. The average contamination levels in poultry feed samples were lower than in maize samples, probably due to the processing or the adding of other ingredients beside maize. As reported by Soriano and Dragacci [25], Silva et al. [26], and Rodríguez-Amaya and Sabino [13] in their reviews on mycotoxins, the distribution of fumonisins is widespread. Compared with other grains, fumonisin contamination of maize is not only more frequent but also accompanied by larger toxin concentrations. The FB_1_ concentrations always exceeded FB_2_ and FB_3_ concentrations; this follows the general pattern of fumonisin contamination in maize and maize-based foods [26, 27]. In the present study, FB_1_ concentrations ranged from 32 to 6,000 *μ*g/kg with a median of 1,300 *μ*g/kg in maize, while this median is 185 *μ*g/kg in poultry feed samples. For FB_2_ and FB_3_ the concentration ranges varied, respectively, from 9 to 2,450 *μ*g/kg and from 7 to 820 *μ*g/kg. The median of total fumonisins (FB_1_ + FB_2_) in maize samples found in the present study (1,840 *μ*g/kg) was below the maximum limit of fumonisins (FB_1_ + FB_2_) recently established by Brazilian regulation [11] for unprocessed maize (5000 *μ*g/kg); only one maize samples analyzed exceed this limit reaching 8760 *μ*g/kg. On the other hand, the poultry feed samples did not exceed the recommended value by the FDA (100,000 *μ*g/kg) [28].

In addition, fully hydrolyzed fumonisin B_1_ (HFB_1_), also named aminopentol (AP_1_), was found in 9% of maize samples. Although numerous fumonisins have been characterized, FB_1_ is usually the most abundant in contaminated foods, except when maize has been treated with base to produce maize flour for tortillas, which hydrolyzes FB_1_ to AP_1_. AP_1_ also appears to have the same liver cancer promoting activity as FB_1_. Heretofore, these in vivo effects of AP_1_ have been somewhat puzzling because AP_1_ is less potent than FB_1_ as an inhibitor of ceramide synthase *in vitro*; AP_1_ is converted to an even more potent metabolite [29]. Our results on the frequency and range of FB_1_ and FB_2_ contaminations in maize are comparable with the data reported by studies conducted in other countries. Sydenham et al. [30] reported a similar incidence of fumonisins in maize meal from USA with slightly lower levels of contamination when compared with our results. It was noted that mean positive values of FB_1_ reaches 1048 *μ*g/kg in USA maize meal and 138 *μ*g/kg in South Africa maize meal, while FB_1_ contaminations in maize reaches 1655 *μ*g/kg in Ghana and 6600 *μ*g/kg in Argentina and Honduras. The same happened in Brazil; when it was observed that all the samples of maize flour from São Paulo State were positive for FB_1_ and FB_2_, mean values were 2100 and 700 *μ*g/kg, respectively [31]. Hirooka et al. [32] investigated maize from great producer regions in Brazil and also found height levels of fumonisins. Almost all samples were positive for FBs. In this work, 16.7% of the analyzed samples had FB_1_ concentration higher than 7500 *μ*g/kg. In contrast to that, there are only few data on contamination of poultry feed samples. However, our results claim for an urgent regulation for fumonisins in Brazil. Cooccurrence of mycotoxins is evident in this study because, besides FBs and BEA that are present in most of samples, other mycotoxins appear as additional contaminants.

Considering the maximum limits established in Brazil or even by the EC for aflatoxins, these toxins were detected in very low levels and only in four samples. The maximum value found for AFB_1_ in maize was 3.0 *μ*g/kg, and media was lower than LOD (nd). AFG_2_ was also found in lower levels in two maize samples and five feed samples. Brazilian regulation establishes maximum limits only for aflatoxins with 20 *μ*g/kg for AFL total (B_1_ + B_2_ + G_1_ + G_2_) in human foods and 50 *μ*g/kg in animal feed, while EC establishes values for AFB_1_ in unprocessed maize of 5 *μ*g/kg and 10 *μ*g/kg for AFL totals. As it can be observed from the work on Brazilian maize and maize-based foods, aflatoxin incidence was low, and the highest contamination levels were founded in Northeast area [12, 13].

The trichothecenes DON and NIV were also found, however, in few samples and in low concentrations, considering the limits established by Brazilian Regulation in cereals of 2,000 *μ*g/kg for DON in 2012 and 1,000 *μ*g/kg in 2016. Biselli and Hummert [33] analyzed DON and T-2 toxins in maize and found an average of 140 and 0.4 *μ*g/kg and maximum levels of 1950 and 8.4 *μ*g/kg, respectively. Concerning other *Fusarium* toxins, zearalenone (ZON) concentration reached 9.80 *μ*g/kg; thus, it did not exceed the norm setting the maximum amount for the mycotoxin at 400 *μ*g/kg in 2012 and 150 *μ*g/kg in 2016. The maximum concentration of moniliformin was 170 *μ*g/kg.

Among the mycotoxins most frequently found in the samples, there was also beauvericin (BEA) which was detected in 96% of maize samples with a media of 12 *μ*g/kg and a maximum of 160 *μ*g/kg and in 92% of feed samples in much lower levels (median of 3.6 *μ*g/kg and maximum of 16.7 *μ*g/kg). Enniatin concentrations in maize samples reached 0.1 *μ*g/kg, 0.3 *μ*g/kg, 5.0 *μ*g/kg, and 1.3 *μ*g/kg for enniatins A, A_1_, B, and B_1_, respectively. This is the first study detecting hexadepsipeptides in Brazilian maize. Uhlig et al. [34] identified this compound group as one of the two with the highest cytotoxicity of the *F. avenaceum* rice culture extracts in PK-15 cells. The cyclic hexadepsipeptides beauvericin (BEA) and enniatins are *Fusarium* secondary metabolites, which are less frequently investigated by routine methods. Beauvericin is toxic to several vertebrate and invertebrate cell cultures, inducing apoptosis, and is known to be a very potent channel-forming molecule inducing pores in biological membranes [35]. Enniatins are known for their phytotoxic and antimicrobial activity. Recently, BEA was found to exhibit phytotoxicity in tomato protoplasts—leading to protoplasts death and decrease in the ascorbate level [36].

Despite a relatively low amount of agroclavine (7.20 *μ*g/kg) found in the samples, this is the first report in which a maize sample presented contamination by this ergot alkaloid. Agroclavine specifically modifies spatial memory in mice by impairing reproduction of conditioned navigation reflex in the Morris water test. This alkaloid modulates activity of the serotonin- and noradrenergic systems of the brain acting as antagonist and partially agonist of 2A-type serotonin receptors (5-HT2A receptors) and as *α*1-adrenoceptor antagonist [37].

Unfortunately, there is only a limited number of surveys concerning *Fusarium* mycotoxins other than fumonisins in poultry feed mixtures, but they clearly show that this kind of feed should be of bigger concern from the mycotoxicological point of view.

Last column of Table 2 shows the factory residue samples contamination where the highest contamination levels were found for almost all toxins. In fact, the cleaning step was important to remove and discard broken maize grains, which were the most contaminated ones, although the mass fraction of this discard was obviously too low to cause any significant decrease of mycotoxins in the remaining grains. Contamination by fumonisins B_1_, B_2_, and B_3_ and BEA occurred in 100% of the samples with a prevalence of FB_1_.

Kojic acid was also detected in 100% of sample reaching concentrations of 344 *μ*g/kg and median 28 *μ*g/kg, respectively. In spite of no adverse effect of kojic acid (KA), its level has been established in chickens at 146 mg/kg in a 21-day feeding study, and the NOAEL (no observed adverse effect level) for thyroid tumor promoting effects of kojic acid has been established at 15.5 mg/kg·day^−1^ in mice and rats [38]. Takizawa et al. [39] provide strong evidence for a tumor-promoting behavior of a 2% KA in a rat diet. At this concentration, KA can be considered as a weak hepatocarcinogenic agent.

## 4. Conclusion

The HPLC-MS/MS method used in this study constituted an alternative to conventional techniques for mycotoxin analysis showing an ultralarge mycotoxin spectra, good sensitivity, rapidness, and applicability to complex matrices such as maize and maize-based feed. It could therefore be applied as routine method for different types of food as well as food production testing. The recovery was between 70 and 120% for 73 mycotoxins in maize while 65 mycotoxins in feed.

Concerning fumonisins, all samples were contaminated, and in some samples, contamination levels exceeded the maximum levels established by the EC. This would lead to increased risk to the consumer health from mycotoxins and emphasizes the urgency for establishing regular monitoring programs for mycotoxins in staple grains in developing countries. The results claim for an urgent regulation for fumonisins in Brazil.

This is the first study dealing with agroclavine, chanoclavine, enniatin A, A_1_, B, beauvericin, and kojic acid contamination of maize and poultry feeds from Brazil. Although some mycotoxin content in maize was low, most samples were contaminated with more than one mycotoxin analyzed. This study suggests that more investigations are needed in this commodity since this survey only covers 2005/2006, and the occurrence may change from year to year implying that further monitoring of mycotoxin in Brazil is justified.

This result reinforces the need to know other mycotoxins in food products to verify the real extension of the mycotoxins in food and feed to protect public health.

## Figures and Tables

**Table 1:** Spiking levels (SL), limit of detection (LOD), and average apparent recoveries of spiked maize and feed.

Toxins	SL	LOD	Recovery (%)	
	*μ*g/kg	*μ*g/kg	Maize	Feed
*Fumonisins *				
Fumonisin B_1_ (FB_1_)	504	8	78 ± 5	51 ± 3
Fumonisin B_2_ (FB_2_)	505	7	76 ± 2	53 ± 2
Fumonisin B_3_ (FB_3_)	50.0	4	98 ± 5	74 ± 7
Hydrolysed fumonisin B_1_ (HFB_1_)	54.9	17	74 ± 5	75 ± 4
*Aflatoxins *				
Aflatoxin B_1_ (AFL B_1_)	25	0.8	71 ± 5	77 ± 3
Aflatoxin G_1 _(AFL G_1_)	25	0.5	80 ± 3	75 ± 6
Aflatoxin B_2_ (AFL B_2_)	25	0.7	83 ± 2	65 ± 7
Aflatoxin G_2_ (AFL G_2_)	25	1	87 ± 2	69 ± 9
*Ochratoxins *				
Ochratoxin A (OTA)	20	1	82 ± 5	94 ± 3
Ochratoxin B (OTB)	20	1	85 ± 5	93 ± 5
Ochratoxin *α* (OT*α*)	11	3	77	84
*Zearalenone *				
Zearalenone (ZON)	100	0.4	86 ± 3	81 ± 3
Zearalenone-4-sulfate	0.4	0.3	86	95
*α*-Zearalenol (*α*-ZOL)	20	3	100 ± 9	90 ± 6
*β*-Zearalenol (*β*-ZOL)	20	4	75 ± 7	69 ± 7
*α*-Zearalenol-glucoside	120	0.8	94 ± 11	99 ± 8
*β*-Zearalenol-glucoside	120	1	110	99
Zearalenone-4-glucoside	20	5	94 ± 19	112 ± 5
*Hexadepsipeptides *				
Beauvericin (BEA)	10	2	66	86
Enniatin A (EA)	0.8	0.1	102 ± 8	87 ± 6
Enniatin A_1_ (EA_1_)	0.56	0.15	100 ± 6	88 ± 9
Enniatin B (EB)	0.53	0.3	66 ± 6	67 ± 13
Enniatin B_1_ (EB_1_)	1.51	0.2	100 ± 3	73 ± 2
Enniatin B_3_ (EB_3_)	0.63	0.04	93	87
*Ergot alkaloids *				
Agroclavine	3.4	0.2	49 ± 7	60 ± 16
Chanoclavine	50	0.4	79 ± 4	80 ± 7
Festuclavine	50	0.15	83 ± 2	70 ± 6
Elymoclavine	50	1	47 ± 4	47 ± 11
Elymoclavine fructoside	50	4	29 ± 4	32 ± 8
Oxidized elymoclavine	50	3	46 ± 3	51 ± 9
Ergine	1.08	0.1	57 ± 4	54 ± 9
Ergotamine	1.08	0.7	24	37
Ergocornine	1.08	1	36	30
Ergocorninine	0.692	0.15	52	62
Ergocristine	1.08	0.3	23	33
Ergocristinine	0.692	0.2	59	61
*α*-Ergocryptine	1.08	0.2	30	36
*α*-Ergocryptinine	0.692	0.1	67	69
Ergometrine	2.17	0.1	90	80
Ergometrinine	0.432	0.07	58 ± 5	43 ± 4
Ergosine	1.08	0.13	37	31
Ergosinine	0.692	0.02	82	48
Dihydroergotamine	1.08	0.5	49	43
Oxidized luol	50	0.3	72	75
Dihydrolysergol	50	0.2	79 ± 2	66 ± 4
Lysergol	50	1	76 ± 2	65 ± 5
*Trichothecenes *				
Deoxynivalenol (DON)	100	20	107 ± 5	99 ± 2
15-Acetyl-deoxynivalenol	50.4	50	104 ± 7	116 ± 18
3-Acetyl-deoxynivalenol	100	20	91 ± 5	96 ± 3
Deoxynivalenol-3-glucoside	20	15	120 ± 11	75 ± 7
Deepoxydeoxynivalenol	25.5	15	127	114
Nivalenol (NIV)	100	50	110 ± 16	90 ± 3
Fusarenon X (F-X)	101	50	100 ± 8	101 ± 5
Toxin HT-2 (HT2)	100	20	99 ± 6	104 ± 4
Toxin T-2 (T2)	100	20	101 ± 2	98 ± 2
Neosolaniol (NEO)	27	3	92 ± 6	95 ± 4
Monoacetoxyscirpenol	10	2	111 ± 16	110 ± 13
Diacetoxyscirpenol	100	1	91 ± 4	98 ± 1
Verrucarol	200	180	80 ± 17	95 ± 8
Verrucarin A	10.7	5	95	91
Roridin A	13.7	1	89	87
T2-Tetraol	42.7	20	76	89
T2-Triol	42.7	20	79	77
*Others *				
Moniliformin (MON)	204	81	87	112
Kojic acid.	300	160	83 ± 3	64 ± 9
Emodin	8.5	4	89	65
Penicillic acid	62.5	20	50 ± 11	39 ± 7
Brefeldin A	62.5	60	95 ± 12	93 ± 7
Roquefortin C	62.5	4	65 ± 5	61 ± 9
Gibberellic acid	85.4	20	102	101
Patulin (PAT)	64.2	100	16	22
Gliotoxin	42.7	12	58	12
Fumitremorgin C	6.4	4	90	79
Altenuene	8.5	6	89	102
Alternariol	17.1	2	91	82
Alternariol monomethyl ether	8.5	0.1	99	81
Sterigmatocystin	8.5	0.4	78	84
Citrinin (CTN)	25.6	30	90	122
Cytochalasin A	62.5	30	18 ± 7	25 ± 8
Cytochalasin B	62.5	10	95 ± 5	89 ± 6
Cytochalasin C	62.5	2	96 ± 11	94 ± 12
Cytochalasin D	62.5	4	102 ± 7	92 ± 4
Cytochalasin H	62.5	30	98 ± 5	96 ± 5
Cytochalasin J	62.5	5	95 ± 5	98 ± 6
Mevinolin	42.7	7	109	65
Mycophenolic acid	23.9	10	106	103
Paxilline	42.7	25	99	66
Penitrem A	12.8	5	133	121
Sulochrin	21.3	4	84	83
Tentoxin	3.39	0.5	148	152
Chaetoglobosin A	21.3	9	10	50
Chetomin	64.0	100	18	17
Meleagrin	21.3	2	92	99
Verruculogen	24.4	50	78	86
Griseofulvin	21.3	10	90	90
Methysergide	0.70	0.4	75	83
Alamethicin-F30	40	3	99	85
HC toxin	43.5	20	87 ± 5	68 ± 4

**Table 2:** Mycotoxins and metabolites detected in maize, poultry feed, and factory residue by liquid chromatography-tandem mass spectrometry (LC-MS/MS). It shows the contamination range, median, and percentage of contaminated samples.

Toxin	Maize				Poultry feed				Factory residue			
	Min. (μg/kg)	Median (μg/kg)	Max. (μg/kg)	%	Min. (μg/kg)	Median (μg/kg)	Max. (μg/kg)	%	Min. (μg/kg)	Median (μg/kg)	Max. (μg/kg)	%
*Fumonisins *												
Fumonisin B_1_ (FB_1_)	32	1,300	6,000	100	50	185	1,118	100	14,085	17,153	27,145	100
Fumonisin B_2_ (FB_2_)	9	540	2,760	99	8	54	474	100	5,927	7,412	10,867	100
Fumonisin B_3_ (FB_3_)	7	190	820	99	nd	27	142	92	1,422	1,853	3,090	100
Fumonisin total (FB_1_ + FB_2_)	41	1,840	8,760	100	58	239	1,592	100	20,012	23,676	36,040	100
Hydrolysed fumonisin B_1_ (HFB_1_)	nd*	6.0	170	9	nd	nd	nd	0	168	366	909	100
*Aflatoxins *												
Aflatoxin B_1_ (AFL B_1_)	nd	nd	3.0	16	nd	nd	nd	0	nd	nd	5.96	44
Aflatoxin B_2_ (AFL B_2_)	nd	nd	nd	0	nd	nd	nd	0	nd	nd	1.10	22
Aflatoxin G_1_ (AFL G_1_)	nd	nd	0.6	1	nd	nd	nd	0	nd	nd	0.52	11
Aflatoxin G_2_ (AFL G_2_)	nd	nd	1.8	4	nd	nd	1,43	14	1.0	1.73	2.51	100
*Trichothecenes *												
Deoxynivalenol (DON)	nd	nd	30.0	4	nd	nd	20	3	nd	nd	nd	0
Nivalenol (NIV)	nd	nd	120.0	5	nd	nd	67	17	nd	nd	nd	0
Zearalenone (ZON)	nd	nd	9.8	12	nd	nd	6.5	39	nd	nd	nd	0
*Hexadepsipeptides *												
Beauvericin (BEA)	nd	12	160	96	nd	3.6	16.7	92	59	116	211	100
Enniatin A (EA)	nd	nd	0.1	1	nd	nd	nd	0	nd	nd	nd	0
Enniatin A_1_ (EA_1_)	nd	nd	0.3	12	nd	nd	0.72	17	nd	nd	0.27	22
Enniatin B (EB)	nd	nd	5.0	34	nd	nd	4.6	78	nd	nd	3.21	44
Enniatin B_1_ (EB_1_)	nd	0.1	1.3	12	nd	nd	12	67	nd	nd	1.12	33
*Others *												
Kojic acid	nd	12	230	65	nd	12	84	67	2.9	28	344	100
Agroclavine	nd	nd	7.2	1	nd	nd	nd	0	nd	nd	nd	0
Chanoclavine	nd	nd	nd	0	nd	nd	nd	0	nd	nd	3.95	33
Moniliformin (MON)	nd	nd	170	8	nd	nd	120	3	nd	220	336	89

*nd (not detected), nd < LOD.
